# Impact of surgery within 6 hours on preoperative rerupture and prognosis of poor-grade aneurysmal subarachnoid hemorrhage

**DOI:** 10.3389/fneur.2026.1824169

**Published:** 2026-07-24

**Authors:** Xiaomei Xu, Qiuyan Shen, Dan Wang, Shibai Sun, Wenwen Che, Yuhai Wang

**Affiliations:** Department of Neurosurgery, No. 904 Hospital of the Joint Logistics Support Force of the Chinese People's Liberation Army, Wuxi, Jiangsu, China

**Keywords:** poor-grade aneurysm, prognosis, rerupture of aneurysm, subarachnoid hemorrhage, timing of operation

## Abstract

**Objective:**

Preoperative rerupture is a major cause of poor outcome in poor-grade aneurysmal subarachnoid hemorrhage (PaSAH). The optimal surgical timing is controversial. This study aims to investigate the effect of surgery within 6 h on preoperative rerupture and the prognosis of PaSAH.

**Methods:**

A retrospective study was performed on 140 PaSAH patients treated at the Neurosurgery Department of the 904th Hospital of the Joint Logistics Support Force between July 2017 and July 2025, with a 6-month postoperative follow-up. Patients were divided into two groups: the surgery within 6 h group and the surgery beyond 6 h group, based on the time from aneurysm rupture to surgery.

**Results:**

Of the 140 patients, 83 (59.3%) were in the surgery within 6 h group, and 57 (40.7%) were in the surgery beyond 6 h group. The surgery within 6 h group had significantly higher proportions of H-H classification, mFisher classification, and decompressive flap use, while the proportion of acute hydrocephalus was notably lower, with statistically significant differences (*p* < 0.05). Compared to the surgery beyond 6 h group, the surgery within 6 h group demonstrated a 17.9% reduction in the preoperative aneurysm rerupture rate (8.4% vs. 26.3%), a 7.5% lower 30-day all-cause mortality rate (24.1% vs. 31.6%), and a 7% higher rate of favorable 6-month outcomes (38.6% vs. 31.6%). After adjusting for acute hydrocephalus, H-H classification, and mFisher classification, the analysis revealed that surgery beyond 6 h significantly increased the risk of preoperative rerupture compared to surgery within 6 h (*RR* = 3.188, 95% *CI* 1.335–7.616, *p* = 0.009). Furthermore, after adjusting for preoperative rerupture, surgery within 6 h significantly improved prognosis (*RR* = 1.145, 95% *CI* 1.030–1.274, *p* = 0.012).

**Conclusion:**

Surgery within 6 h effectively reduces the risk of preoperative rerupture in PaSAH patients and significantly improves long-term outcomes.

## Introduction

Poor-grade aneurysmal subarachnoid hemorrhage are defined by the Hunt-Hess classification or the World Federation of Neurosurgical Societies (WFNS) classification IV-V, representing approximately 18–24% of all aneurysmal subarachnoid hemorrhage (aSAH) cases (1). According to reports, the mortality rate for these aneurysms is 26%, and the disability rate is 60% (2). Due to their critical onset and high mortality and disability, they remain a focal point of neurosurgical research.

There is ongoing debate regarding the effect of surgical timing on the prognosis of aSAH patients. Most studies suggest that ultra-early and early surgical interventions benefit patients with poor-grade aSAH (PaSAH) by improving outcomes (3–5), although some argue that surgical timing does not significantly impact prognosis in PaSAH patients (6). Furthermore, studies report an aneurysm rerupture and bleeding rate of 7–11%, with the risk increasing with the aneurysm grade (7). Rerupture, once it occurs, can rapidly exacerbate the patient’s condition and significantly worsen prognosis. The clinical value of surgery within 6 h of ictus in patients with poor-grade aneurysmal subarachnoid hemorrhage remains unclear.

This study aims to retrospectively analyze the clinical data of 140 PaSAH patients who underwent clipping surgery at the 904th Hospital of the Joint Logistics Support Force from July 2017 to July 2025, providing a clinical foundation for optimal treatment timing in PaSAH cases.

## Materials and methods

### Data source and population

A retrospective analysis was conducted on clinical data from 140 PaSAH patients treated at the Neurosurgery Department of the 904th Hospital of the Joint Logistics Support Force from July 2017 to July 2025. Inclusion criteria were as follows: (1) Age 18–75 years; (2) Subarachnoid hemorrhage (SAH) or intracerebral hemorrhage (ICH) due to aneurysm rupture, confirmed by cranial CT, CTA, or DSA; (3) Hunt-Hess grade IV-V for aSAH; (4) aSAH treated with surgical clipping. Exclusion criteria were as follows: (1) History of head disease or head surgery; (2) Severe cardiac, pulmonary, or other systemic disorders; (3) Incomplete case data or loss to follow-up; (4) Treatment withdrawal due to family reasons; (5) Patients who only received external ventricular drainage (EVD) or decompressive craniotomy without aneurysm clipping; (6) Unclear aneurysm rupture to surgery duration. This study was approved by the hospital’s Ethics Review Committee (No: 20251115).

### Grouping of operative timing and procedures

The time from aneurysm rupture to the start of surgical clipping was defined as the operative timing, with the rupture time confirmed by the patient’s onset time of symptoms. Patients were stratified according to the time from onset to surgery: the surgery within 6 h group included 83 patients, while the surgery beyond 6 h group included 57 patients.

Craniotomy for aneurysm clipping was conducted under general anesthesia, with the surgical approach chosen based on aneurysm location. Considering the high risk of intracranial hypertension in patients with poor-grade aneurysms, surgical incisions were designed to accommodate potential intraoperative decompressive craniectomy, and incisions for preoperative EVD were marked concurrently if necessary. For patients with high intracranial pressure (ICP) or acute hydrocephalus on preoperative CT, EVD catheters were placed first, and cerebrospinal fluid (CSF) was released to lower ICP to less than 40 mmHg prior to craniotomy. After bone flap removal and dural opening, intermittent slow CSF drainage via EVD was adopted when ICP exceeded 20 mmHg to optimize surgical exposure. The parent artery was exposed via arachnoid dissection, followed by aneurysm neck dissection. Temporary parent artery occlusion was applied selectively, and permanent aneurysm clips were used to obliterate the aneurysm neck. Complete aneurysm clipping was confirmed by aneurysmal dome puncture, intraoperative indocyanine green angiography or Transcranial Color Doppler Ultrasound, and vascular stenosis of parent and distal arteries was screened simultaneously. The subarachnoid space was thoroughly irrigated with normal saline to clear hemorrhagic fluid. After adequate hemostasis, whether to perform decompressive craniectomy was determined by pre-closure ICP, followed by routine scalp suturing.

### Treatment protocol

Upon arrival at the emergency department, the patient’s condition was assessed, and vital signs were stabilized. Once stable, emergency cranial CT and CTA or DSA were performed for further diagnostic confirmation. Routine preoperative laboratory tests were conducted for patients with stable vital signs. Upon family consent and signed informed consent forms, emergency aneurysm clipping was performed. Patients who either refused surgery or had unstable vital signs were admitted to the NICU for conservative treatment. Patients with acute hydrocephalus underwent extracranial drainage prior to clipping, with the procedure deferred until the patient consented or vital signs stabilized. Postoperatively, patients were transferred to the NICU for continued management, with an emphasis on blood pressure and ICP control, alongside medications such as nimodipine to prevent vasospasm.

### Observation indicators

Data were collected through a retrospective chart review using a standardized data abstraction form. After retrieving the original imaging data and electronic medical records, two investigators independently re-reviewed the images to assess and document key clinical and imaging parameters. The recorded datasets were then compared for consistency, and any discrepancies or ambiguous entries were resolved through discussion and consensus; if necessary, a third senior investigator was consulted. This process was implemented to enhance data reliability and minimize errors inherent to retrospective reviews.

(1) General data: gender, age, hypertension history, diabetes history.
(2) Disease-related data: Hunt-Hess classification, modified Fisher classification, aneurysm location, preoperative pupillary changes, and surgical approach, acute hydrocephalus, intraventricular hematoma, intracranial hematoma, EVD, craniectomy, postoperative shunt-dependent hydrocephalus, cerebral vasospasm, delayed cerebral ischemia, and intracranial infection.
(3) Outcome data: preoperative rerupture rate, 30-day all-cause mortality, and 6-month postoperative follow-up.

### Follow-up

The GOS score was used to evaluate the prognosis of patients with 6 months after the operation. All patients were regularly followed up by professional staff through outpatient service and telephone, and GOS score assessment results were determined: GOS score of 4–5 indicated good prognosis, 1–3 indicated poor prognosis. This study initially screened 149 patients with PaSAH; however, 9 cases were excluded due to loss to follow-up, resulting in a final cohort of 140 participants. The follow-up completeness rate was 93.96%.

### Statistical analysis

Data analysis was performed using SPSS 25. Categorical variables were expressed as frequencies and percentages (*n* (%)), and A one-way analysis was conducted first, followed by adjusted Poisson regression for variables with *p* < 0.05 to calculate the relative risk (*RR*) and 95% confidence interval (*CI*). A *p*-value < 0.05 was considered statistically significant.

## Results

**Figure 1:**
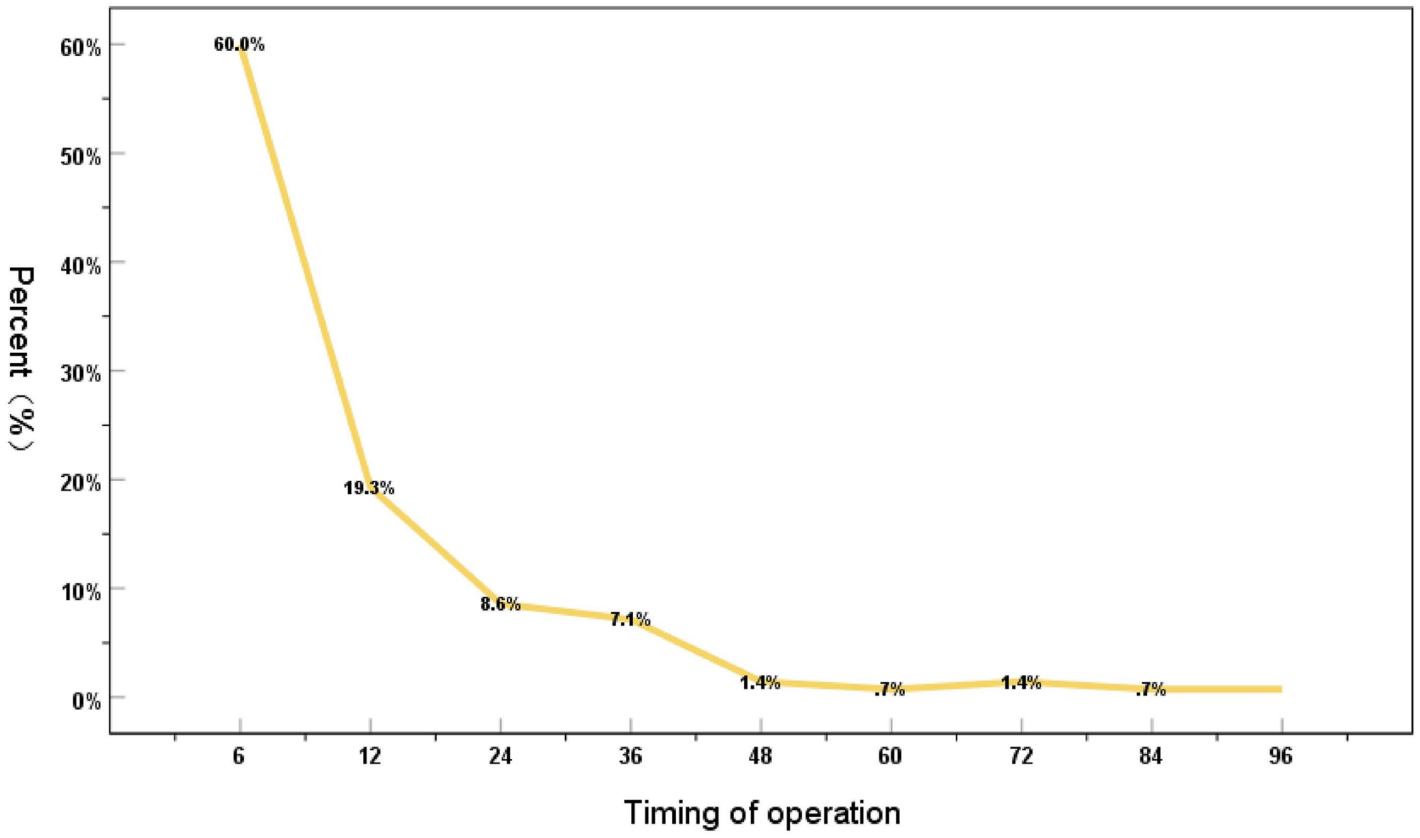
Distribution of hours from onset to surgery for all patients diagnosed with PaSAH.

Among the 140 PaSAH patients, 54 were male, and 86 were female. The mean age of the cohort was 57 ± 12 years (Table 1). The operative window ranged from 1 to 96 h. The surgery within 6 h group included 83 patients (59.3%), while the surgery beyond 6 h group comprised 57 patients (40.7%). Detailed distribution is shown in Figure 1.

**Table 1:** Comparison of baseline data between the two groups.

Variables	Total (*N* = 140)	Surgery within 6 h (*n* = 83), *n* (%)/M	Surgery beyond 6 h (*n* = 57), *n* (%)/M	*χ^2^*	*p*
Gender				0.507^a^	0.477
Male	54	30 (36.1)	24 (42.1)		
Female	86	53 (63.9)	33 (57.9)		
Age				3.537^a^	0.060
< 60	82	54 (65.1)	28 (49.1)		
≥ 60	58	29 (34.9)	29 (50.9)		
Hypertension history	110	68 (82.9)	42 (73.7)	1.740^a^	0.187
Diabetes history	13	9 (11.0)	4 (7.0)	0.621^a^	0.431
H-H grade				5.082^a^	0.024
IV	65	32 (38.6)	33 (57.9)^*^		
V	75	51 (61.4)	24 (42.1)^*^		
mFisher grade				9.546^b^	0.004
1	1	0 (0.0)	1 (1.8)^**^		
2	0	0 (0.0)	0 (0.0)^**^		
3	30	11 (13.6)	19 (33.9)^**^		
4	106	70 (86.4)	36 (64.3)^**^		
Aneurysm rerupture	22	7 (8.4)	15 (26.3)^**^	8.159^a^	0.004
Aneurysm location				5.558^a^	0.235
ACoAA	38	23 (27.7)	15 (26.3)		
PCoAA	28	17 (20.5)	11 (19.3)		
MCAA	44	30 (36.1)	14 (24.6)		
Other ACA	19	7 (8.4)	12 (21.1)		
PCA	11	6 (7.2)	5 (8.8)		
Dilated pupil				3.086^a^	0.214
YES					
Unilateral	30	16 (19.3)	14 (24.6)		
Bilateral	24	18 (21.7)	6 (10.5)		
NO	86	49 (59.0)	37 (64.9)		
Surgical approach				0.247^a^	0.619
Pterional approach	135	79 (95.2)	56 (98.2)		
Suboccipital approach	5	4 (4.8)	1 (1.8)		
DC	87	66 (79.5)	21 (42.0)^**^	19.413^a^	< 0.001
Acute hydrocephalus	43	20 (24.1)	23 (40.4) ^*^	4.196^a^	0.041
IVH	55	32(38.6)	23(40.4)	0.046^a^	0.831
EVD	128	75 (90.4)	53 (93.0)	0.056^a^	0.813
ICH	93	57 (68.7)	36 (63.2)	0.461^a^	0.497
SDHC	38	24 (28.9)	14 (25.0)	0.258^a^	0.611
Cerebral vasospasm	22	13 (15.9)	9 (15.8)	0.000^a^	0.992
Cerebral ischemia	72	42 (51.2)	30 (52.6)	0.027^a^	0.870
Intracranial infection	26	14 (16.9)	12 (21.1)	0.391^a^	0.532
Epilepsy	23	16 (19.3)	7 (12.5)	1.112^a^	0.292

a Chi-square test.

b Fisher’s exact test.

^**, *^p < 0.01 and *p* < 0.05, compared to the Surgery beyond 6 h group. H-H grade, Hunt-Hess grade; mFisher scale, modified Fisher grade; ACoAA, anterior communicating artery aneurysm; PCoAA, posterior communicating artery aneurysm; MCAA, middle cerebral artery aneurysm; ACA, anterior circulation aneurysm; PCA, posterior circulation aneurysm; DC, decompressive craniectomy; IVH, intraventricular hemorrhage; EVD, external ventricular drainage; ICH, intracerebral hematoma; SDHC, shunt-dependent hydrocephalus.

### Univariate analysis of surgical timing in the two groups

The surgery within 6 h group had significantly higher proportions of H-H grade V (61.4%, 51/83 vs. 42.1%, 24/57), mFisher grade 4 (84.3%, 70/83 vs. 63.1%, 36/57), and craniotomy for decompression (79.5%, 66/83 vs. 36.8%, 21/57) compared to the surgery beyond 6 h group. Conversely, the proportion of acute hydrocephalus was significantly lower in the surgery within 6 h group (24.1%, 20/83 vs. 40.4%, 23/57), with statistically significant differences (*p* < 0.05). No significant differences were found between the two groups regarding gender, age, history of hypertension, history of diabetes, aneurysm location, surgical approach, intraventricular hematoma, intracranial hematoma, EVD placement, shunt-dependent hydrocephalus, cerebral vasospasm, cerebral ischemia, preoperative mydriasis, intracranial infection, or epilepsy (Table 1).

### Comparison of outcome data between the two groups

**Figure 2:**
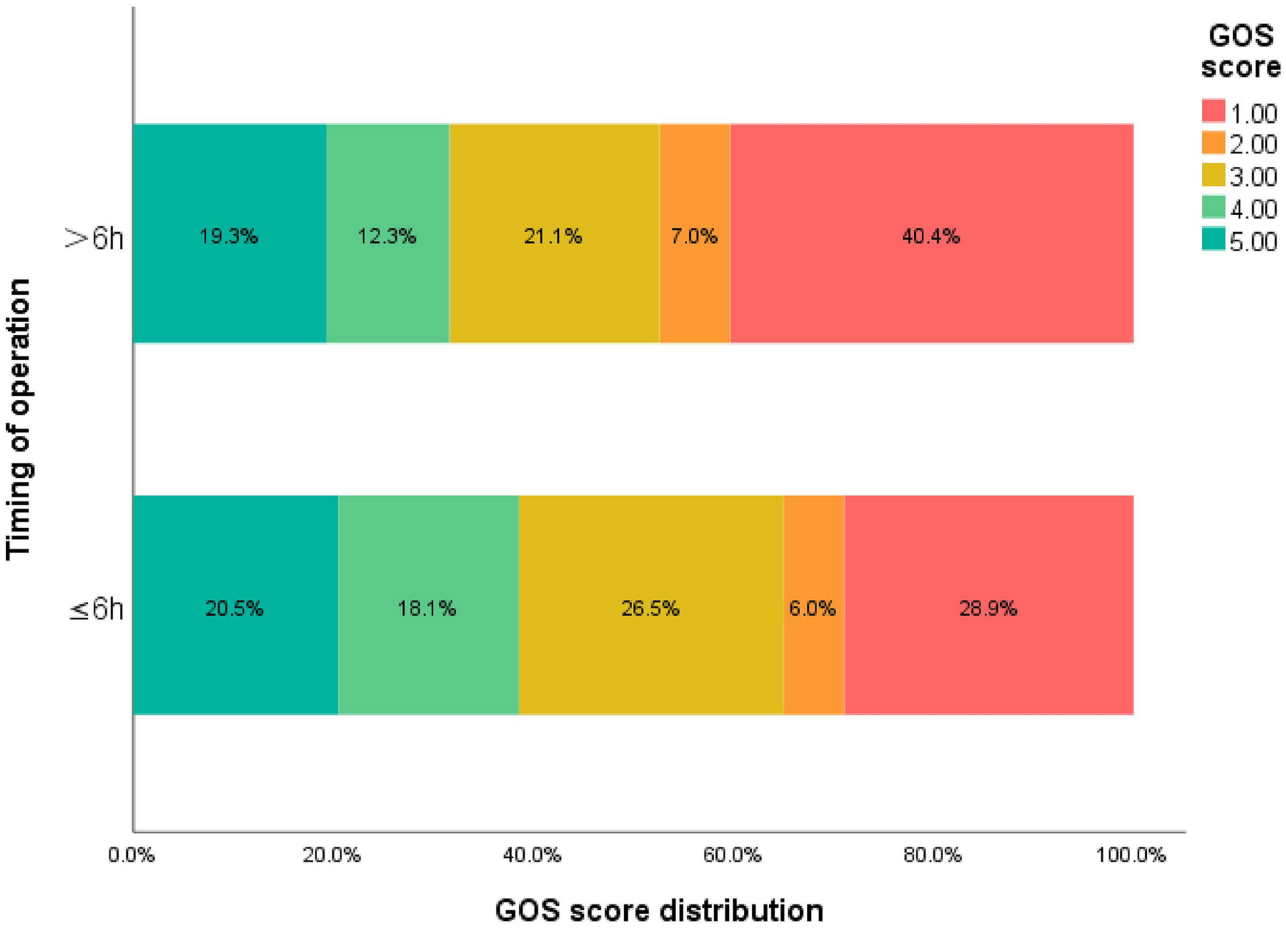
Distribution of GOS scores at different surgical timings.

The aneurysm rerupture rate in the surgery within 6 h group (8.4%, 7/83) was 17.9% lower than in the surgery beyond 6 h group (26.3%, 15/57), with statistically significant differences (*p* < 0.01). The 30-day all-cause mortality rate was 7.5% lower in the surgery within 6 h group (24.1%, 20/83) compared to the surgery beyond 6 h group (31.6%, 18/57), however, the difference was not statistically significant. Furthermore, the 6-month favorable prognosis rate in the surgery within 6 h group (38.6%, 32/83) was 7% higher than in the surgery beyond 6 h group (31.6%, 18/57), however, the difference was not statistically significant (Table 2 and Figure 2).

**Table 2:** Comparison of outcome data between the two groups.

Variables	Total (*N* = 140)	Surgery within 6 h (*n* = 83), *n* (%)/M	Surgery beyond 6 h (*n* = 57), *n* (%)/M	*χ^2^*	*p*
Aneurysm rerupture	22	7 (8.4)	15 (26.3)^**^	8.159	0.004
30-day all-cause mortality	38	20 (24.1)	18 (31.6)	0.957	0.328
6-month GOS				0.716	0.397
Favorable (4–5 score)	50	32 (38.6)	18 (31.6)		
Unfavorable (1–3 score)	90	51 (61.4)	39 (68.4)		

GOS, Glasgow Outcome Scale; ^**^p < 0.01, compared to the Surgery beyond 6 h group.

### Modified Poisson regression analysis of the influence of operation time on the rerupture

Using aneurysm rerupture as the dependent variable, a modified Poisson regression model was established with variables from univariate analysis (*p* < 0.05) as independent variables. Among these, the decompressive craniectomy variable corresponded to procedures performed after aneurysm rerupture, so this variable was excluded from the analysis. After adjusting for acute hydrocephalus, H-H classification, and mFisher classification, the results indicated that surgery beyond 6 h significantly increased the risk of preoperative rerupture compared to surgery within 6 h (*RR* = 3.188, 95% *CI* 1.335–7.616, *p* = 0.009) (Table 3).

**Table 3:** Modified Poisson regression model for identifying risk factors for aneurysm rerupture.

Variables	*B*	Wald	*RR* (95% *CI*)	*p*
Acute hydrocephalus	0.870	4.673	2.387 (1.085–5.255)	0.031
H-H grade	0.433	1.209	1.542 (0.713–3.337)	0.272
mFisher grade	0.482	1.023	1.619 (0.636–4.120)	0.312
Timing of operation	1.160	6.812	3.188 (1.335–7.616)	0.009

H-H grade, Hunt-Hess grade; mFisher scale, modified Fisher grade.

### Modified Poisson regression analysis on the influence of operation time on prognosis

Similarly, using poor prognosis as the dependent variable, this study established a modified Poisson regression model that included *p* < 0.05 indicators from univariate analysis and preoperative recurrent hemorrhage as independent variables. After adjusting for acute hydrocephalus, craniotomy decompression, preoperative rerupture, H-H grade, and mFisher grade, the results revealed that surgery beyond 6 h significantly increased the risk of poor prognosis compared to surgery within 6 h (*RR* = 1.145, 95% *CI* 1.030–1.274, *p* = 0.012) (Table 4).

**Table 4:** Modified Poisson regression model for identifying risk factors for prognosis.

Variables	*B*	Wald	*RR* (95% *CI*)	*p*
Acute hydrocephalus	0.044	0.906	1.045 (0.955–1.143)	0.341
Aneurysm rerupture	0.015	0.063	1.015 (0.902–1.142)	0.802
DC	0.161	5.957	1.174 (1.032–1.336)	0.015
H-H grade	0.154	8.235	1.167 (1.050–1.297)	0.004
mFisher grade	0.028	0.172	1.028 (0.901–1.175)	0.679
Timing of operation	0.135	6.240	1.145 (1.030–1.274)	0.012

DC, decompressive craniectomy; H-H grade, Hunt-Hess grade; mFisher scale, modified Fisher grade.

## Discussion

Advancements in modern diagnostic and therapeutic technologies have led to certain improvements in the clinical prognosis of PaSAH compared to earlier studies. However, the overall mortality rate remains as high as 40–65%, with a poor prognosis rate of 70% (8–13). If PaSAH is not managed promptly, the risk of rerupture within 24 h of onset can reach 15–20%, with rerupture being a leading cause of death and disability. As a result, early surgical intervention is widely supported in current literature for PaSAH patients (14–16).

### Surgical timing for poor-grade aneurysmal subarachnoid hemorrhage

There is no consensus regarding the optimal timing for surgical intervention in PaSAH. Existing guidelines categorize the timing into four approaches: ultra-early surgery (within 24 h of aneurysm rupture), early surgery (24–72 h post-hemorrhage), late surgery (3–10 days post-hemorrhage), and delayed surgery (after 10 days) (3–5, 17–19). Some studies highlight significant perioperative risks and a high incidence of complications associated with microsurgical procedures performed within the first 2 weeks (17). Other reports suggest that early intervention may be beneficial for PaSAH patients (3–5, 18), while surgery between 3 and 10 days post-hemorrhage may elevate the risk of poor prognosis (18). Guidelines generally recommend early treatment of ruptured aneurysms to reduce rerupture rates following aSAH (15).

The definition of “optimal timing” for ultra-early surgery remains inconsistent. Most studies define it as surgery within 24 h of SAH (6, 20), while others extend the window to 48 h (13, 18, 21). This approach aims to promptly address intracranial aneurysms, thereby reducing complications such as rerupture, cerebral vasospasm, and cerebral edema. It is particularly crucial for PaSAH patients, who face higher risks of early rerupture and poorer prognoses, making earlier surgical intervention more beneficial.

The rerupture rate of PaSAH is approximately 20%, compared to only 5% in low-grade aSAH patients (22). Studies also suggest that nearly all rerupture cases occur within 24 h, with 83% occurring within the first 12 h (23). This highlights the fact that treatment initiated within 24 h is not sufficiently early, and earlier intervention may significantly reduce the risk of recurrence. Previous literature has reported that the period of 2 to 12 h after the onset of PaSAH is a high-risk window for rerupture, with 6 h as the optimal balance point for risk prevention and preoperative preparation (23–25). Meta-analyses demonstrated that surgery performed within 6 h could effectively reduce the incidence of rerupture and improve the rate of favorable neurological outcomes in patients with aSAH, without increasing surgical complications (26). Accordingly, we adopted ≤6 h after symptom onset as the surgical cutoff value for PaSAH in the present study. To investigate whether surgery within 6 h can reduce the risk of preoperative aneurysm rerupture, recent practice at our institution has involved performing surgery within 6 h after aneurysm rupture upon obtaining informed consent from the patient’s family, substantially advancing the timing of surgical intervention.

### Effect of surgery within 6 h on rerupture of poor-grade aneurysm

The results of this study demonstrated that only 7 of the 83 patients in the surgery within 6 h group experienced aneurysm rerupture before surgery, yielding a rerupture rate of 8.4%. In contrast, 15 of the 57 patients in the surgery beyond 6 h group had aneurysm rerupture prior to surgery, with a rerupture rate of 26.3%. Among the 15 patients in the surgery beyond 6 h group with rerupture, 11 had rerupture within 7–24 h after the initial hemorrhage. These findings revealed a 17.9% reduction in the preoperative aneurysm rerupture rate in the surgery within 6 h group compared to the surgery beyond 6 h group (*RR* = 3.188, 95% *CI* 1.335–7.616, *p* = 0.009), indicating that surgery within 6 h significantly reduced the risk of preoperative aneurysm rerupture.

However, there are critics of ultra-early surgery, who argue that it is challenging due to brain swelling often present in the early stages of severe SAH, coupled with elevated ICP. Surgery performed under these conditions may increase the risk of brain tissue damage (17, 22).

For PaSAH patients with high ICP, several measures were employed: (1) EVD was placed on the ipsilateral side of the operation before or during surgery, and cerebrospinal fluid was gradually released during the procedure to reduce ICP; (2) A large bone flap craniotomy was performed to open the dura mater and further alleviate ICP; (3) Osmotic diuretics, such as mannitol, were administered to reduce intracellular fluid compartment during surgery. These measures effectively lowered ICP during surgery, facilitating the procedure. This is also the reason why more than 90% of patients received an EVD, despite the IVH incidence in both groups of this study being below 40%. Additionally, 75 of the 83 patients in the surgery within 6 h group received EVD before or during surgery, and no cases of rebleeding due to EVD were observed. The gradual release of cerebrospinal fluid during surgery not only reduced ICP but also facilitated intraoperative aneurysm identification and clipping. Furthermore, it helped prevent acute hydrocephalus, reduced postoperative cerebral edema, and improved patient outcomes. Furthermore, patients in this group who underwent surgery within 6 h presented with more severe conditions, and a higher proportion exhibited elevated intracranial pressure at the time of procedure completion; consequently, a greater proportion required intraoperative decompressive craniectomy.

### Impact of surgery within 6 h on the prognosis of poor-grade aneurysmal subarachnoid hemorrhage

Compared to the surgery beyond 6 h group, the 30-day all-cause mortality rate was reduced by 7.5%, while the good prognosis rate increased by 7% (*RR* = 1.145, 95% *CI* 1.030–1.274, *p* = 0.012), significantly improving patient outcomes. This improvement is likely attributed to a reduced risk of rerupture and acute hydrocephalus.

The mortality rate due to rerupture in PaSAH can be as high as 70%, with earlier rerupture correlating with worse prognosis (3, 18). In this study, among the 7 patients in the surgery within 6 h group who experienced aneurysm rerupture, 1 patient’s condition worsened from Grade II to Grade IV before surgery due to rerupture, and 3 patients’ conditions worsened from Grade IV to Grade V. Additionally, 3 patients with Grade V conditions had further deterioration. Of these, 3 patients died, 1 survived in a vegetative state, 2 were severely disabled, and 1 was moderately disabled. The rerupture mortality rate in this group was 42.9%, and the poor prognosis rate was 85.7%. Among the 15 patients in the surgery beyond 6 h group with rerupture, 1 patient’s condition worsened from Grade II to Grade IV, 3 patients worsened from Grade II to Grade V, 3 patients progressed from Grade III to Grade V, and 5 patients deteriorated from Grade IV to Grade V. This led to 10 deaths, 2 severely disabled patients, and 3 patients with favorable outcomes (all of whom had acute hydrocephalus preoperatively). The rerupture mortality rate in the surgery beyond 6 h group was 66.7%, with an 80% poor prognosis rate. These results suggest that aneurysm rerupture significantly impacts the prognosis of PaSAH patients.

The improved prognosis associated with surgery within 6 h may not only stem from its effectiveness in reducing the risk of rerupture but also from its potential to lower the incidence of acute hydrocephalus. Acute hydrocephalus is common in PaSAH and can further exacerbate the patient’s condition (27–33). EVD positively impacts the prognosis of patients with acute hydrocephalus (34). In this study, the incidence of acute hydrocephalus in the surgery within 6 h surgery group was 24.1%, which was 16.3% lower than the 40.4% incidence in the surgery beyond 6 h group (*p* < 0.05).

### Vasospasm and cerebral ischemia

The incidence of vasospasm was below 16% while cerebral ischemia occurred in more than 50% of patients in both groups, revealing that macrovascular vasospasm was not the dominant cause of cerebral ischemia or infarction among patients with PaSAH. Cerebral ischemia following SAH is mediated by multiple mechanisms, among which early brain injury, microcirculatory dysfunction and neuroinflammation play more critical roles than isolated macrovascular vasospasm (35). Blood degradation products after aneurysm rupture cause microvascular endothelial injury, pericyte dysfunction and diffuse microthrombosis, impairing cerebral autoregulation and inducing focal hypoperfusion, which can trigger cerebral ischemia without significant macrovascular vasospasm (36). Excessive neuroinflammation and oxidative stress disrupt the blood–brain barrier, aggravate microvascular occlusion and neuronal injury, and drive the progression of vasospasm-independent cerebral ischemia (37). Acute intracranial hypertension, early global cerebral hypoperfusion and perioperative hemodynamic fluctuations commonly seen in PaSAH patients further worsen these pathological injuries, which explains our clinical finding of low vasospasm but markedly elevated cerebral ischemia incidence.

### Postoperative intracranial infection

Intracranial infection occurred in over 15% of patients in both groups, considerably higher than the 0.5–5% infection rate of routine intracranial aneurysm operations (38). The high infection rate was primarily related to the large proportion of PaSAH treated via emergency surgery. Emergency settings precluded adequate preoperative skin preparation, prophylactic antibiotics and infectious screening; preoperative aspiration and latent pulmonary infection might also lead to hematogenous intracranial infection (39). EVD was commonly required in PaSAH patients, and prolonged indwelling catheter use markedly elevated infection risk. Combined with prolonged coma, stress-related immunosuppression and malnutrition, patients’ immune defense against infection was compromised. The cumulative effect of these multiple risk factors explained the higher infection rate observed herein relative to previous studies focusing on elective and low-grade aneurysm surgeries (39).

### Limitations

This study has several limitations. 1. As a single-center retrospective study, inherent selection bias exists. Surgical timing was non-random and depended on operating room availability, surgeon judgment, transfer delays, and family consent speed. Critically ill patients with severe neurological deterioration were preferentially offered earlier surgery, resulting in significantly higher Hunt-Hess V grade, mFisher grade, and decompressive craniectomy rates in the within-6-h group. Although multivariate adjustment was performed, residual confounding bias could not be completely eliminated, and unequal case numbers between groups may affect statistical stability. 2. Patients undergoing surgery beyond 6 h were not further stratified for subgroup analysis. 3. The lack of significant between-group differences in delayed cerebral ischemia may be related to the limited sample size. 4. Only surgical cases were included, while endovascular treatment cases were excluded, which may limit the generalizability of the results.

## Conclusion

The findings of this study suggest that surgery within 6 h can effectively reduce preoperative rerupture and the incidence of acute hydrocephalus in PaSAH patients, leading to a significant improvement in prognosis. However, the impact on the development of delayed cerebral ischemia requires further investigation.

## Data Availability

The raw data supporting the conclusions of this article will be made available by the authors, without undue reservation.
